# Refractory Diabetic Macular Edema

**Published:** 2010-07

**Authors:** James Bainbridge

**Affiliations:** Moorfields Eye Hospital and UCL Institute of Ophthalmology, London, UK

Diabetic macular edema (DME) is arguably one of the most important challenges in ophthalmology. Macular edema refractory to laser photocoagulation remains the most prevalent cause of untreatable vision loss in diabetes and is responsible for visual disability in millions of people worldwide. The lack of an effective therapeutic solution accounts for the range of interventions proposed. These include intraocular delivery of corticosteroids and anti-VEGF antibodies, and the surgical alternative of vitrectomy with or without removal of the internal limiting membrane (ILM). In this issue of Journal of Ophthalmic & Vision Research, Dehghan and co-authors^1^ present the results of a prospective interventional case series of triamcinolone-assisted vitrectomy with ILM peeling for refractory diffuse non-tractional DME. They found no significant effect on visual acuity despite a reduction in mean macular thickness. The publication of studies such as this helps redress the biases that tend to promote the reporting and publication of positive findings. This study also illustrates the challenges associated with designing an experiment that will allow clear conclusions to be drawn about the value of combination treatments in such a complex condition.

A number of series have suggested that vitrectomy alone can improve macular function in DME. The rationale for this approach involves relief of mechanical tangential or antero-posterior tractional forces associated with a taut thickened posterior hyaloid at the macula. There is consensus that DME associated with such traction, evident on biomicroscopy or optical coherence tomography (OCT), can benefit from vitrectomy with removal of the attached posterior hyaloid membrane. Removal of the ILM, which can itself be thickened in diabetic eyes, has been proposed as an additional procedure to ensure complete removal of cortical vitreous, and to reduce the likelihood of subsequent epiretinal membrane formation by inhibiting migration and reproliferation of astrocytes.

Whether vitrectomy can improve vision in eyes with DME but no evidence of vitreomacular traction, however, has not been established. The rationale for vitrectomy in this context is certainly less obvious but possible benefits include improved retinal oxygenation by promotion of intraocular fluid currents, and relief of any subclinical tractional forces. Removal of the ILM in non-tractional refractory DME might favorably alter hydrostatic forces across the inner retina and/or stimulate beneficial, if short-lived, inflammatory responses. Few studies have been able to determine with confidence the efficacy and adverse effects of these interventions and we have to rely on non-randomized and poorly controlled case series that are frequently confounded by the adjunctive use of triamcinolone and/or macular photocoagulation. However, a recent randomized controlled trial of vitrectomy with either ILM peeling or intravitreal triamcinolone for diffuse non-tractional DME by Figueroa and coworkers^2^ identified no sustained anatomical or functional improvement. In their report, Dehghan et al^1^ describe their experience with vitrectomy and ILM peeling in similar eyes. They acknowledge that the adjunctive use of triamcinolone could have contributed to the observed outcome, and the lack of a control group means that the results cannot be compared with the natural course of the condition or any standard treatment. Despite these limitations, however, their conclusions are consistent with those of Figueroa et al in identifying no clear benefit in terms of visual acuity. The weight of available evidence suggests that vitrectomy for non-tractional DME does not offer significant benefit in terms of visual acuity, regardless of whether the ILM is removed.

Dehghan et al highlight in their work a discrepancy between anatomical and functional results, one that is frequently apparent following interventions for DME. Studies on both vitrectomy and local administration of corticosteroids have consistently identified a significant reduction in retinal thickness with no detectable or minimal benefit on visual acuity in many eyes. While this discrepancy might be explained in some series by the development of cataract, similar findings in pseudophakic eyes question the extent to which the functional deficit in DME is reversible. In the near future, we can expect that developments in structural and functional retinal imaging will facilitate a more detailed characterization of macular disease and address this question with a better understanding of the impact of therapeutic interventions. For example, spectral domain high-resolution OCT is likely to help identify eyes in which thickening of the posterior hyaloid and ILM could respond favorably to surgical intervention more reliably and at an early stage of the disease. Multispectral imaging will enable non-invasive characterization of cellular and molecular processes in DME to help clarify its pathogenesis and develop better treatments.

We should continue to question our narrow focus on visual acuity as a sole outcome measure of macular function. Other techniques such as microperimetry and multifocal electroretinography together with visual function questionnaires can provide highly relevant information that could more sensitively detect changes in macular function than can be demonstrated by testing visual acuity alone. With the benefit of better insight into the relationship between macular structure and function we can hope to intervene promptly and effectively, with the aim of making refractory DME a thing of the past.
